# Supplementary material for: Global floating kelp forests have limited protection despite intensifying marine heatwave threats
Source: Nat Commun. 2025 Apr 3;16:3173. doi: 10.1038/s41467-025-58054-4 (PMC11968876; doi:10.1038/s41467-025-58054-4)
Supplement: Supplementary file 1 — Supplementary Information [file 41467_2025_58054_MOESM1_ESM.pdf]

Supplementary Information

Global floating kelp forests have limited protection despite intensifying marine heatwave threats

Authors: Nur Arafeh-Dalmau, Juan Carlos Villaseñor-Derbez, David S. Schoeman, Alejandra Mora-Soto, Tom W. Bell, Claire L. Butler, Maycira Costa, Loyiso V. Dunga, Henry F. Houskeeper, Cristian Lagger, Carolina Pantano, Daniela Laínez del Pozo, Kerry J. Sink, Jennifer Sletten, Timothe Vincent, Fiorenza Micheli, Kyle C. Cavanaugh

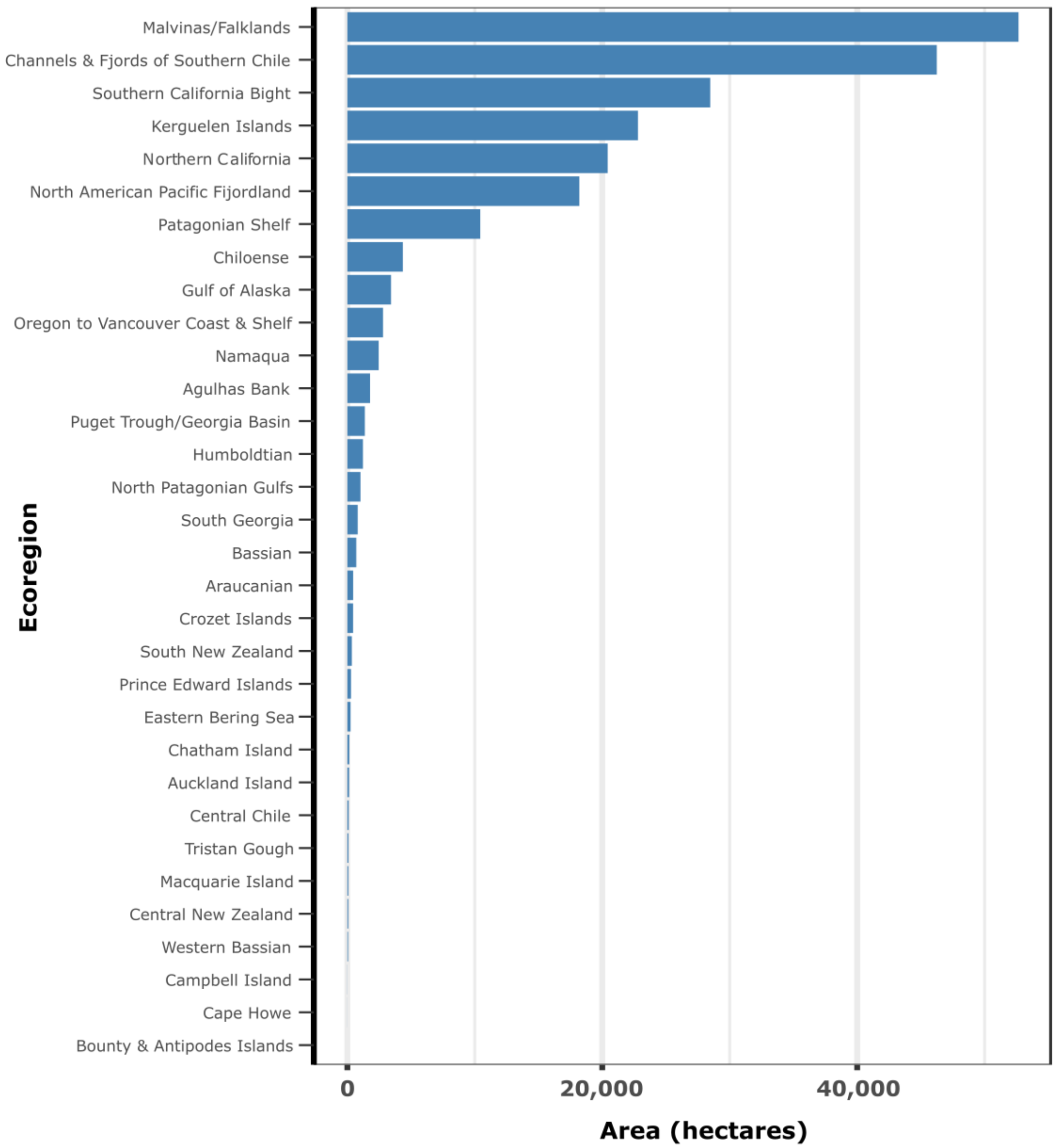

Supplementary Figure 1.- Area of floating kelp forest (hectares) by ecoregion based on remote sensing maps.

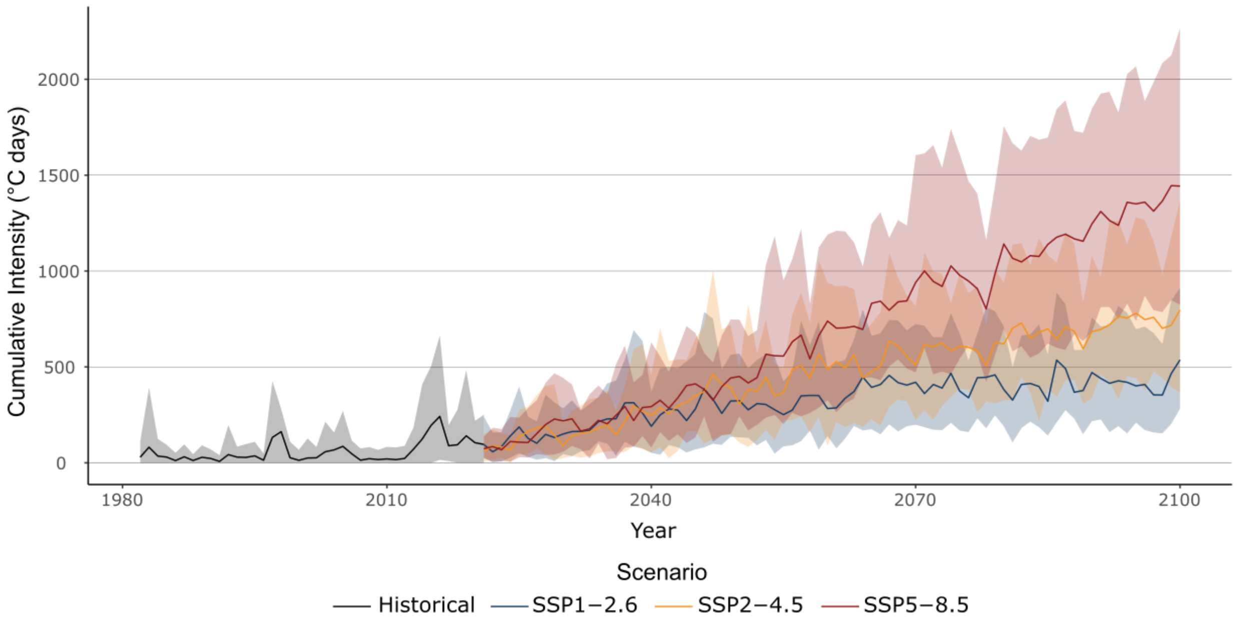

Supplementary Figure 2.- Global exposure of floating kelp forests to contemporary and future marine heatwaves. Global exposure of kelp forest to historical (1982-2020) and future cumulative annual MHW intensities (2021-2100) across three climate scenarios (SSP-1.26, SSP-2.45, SSP-5.85). The solid line shows the mean across ensemble medians for all pixels, and the shaded area represents the 5th and 95th percentiles.

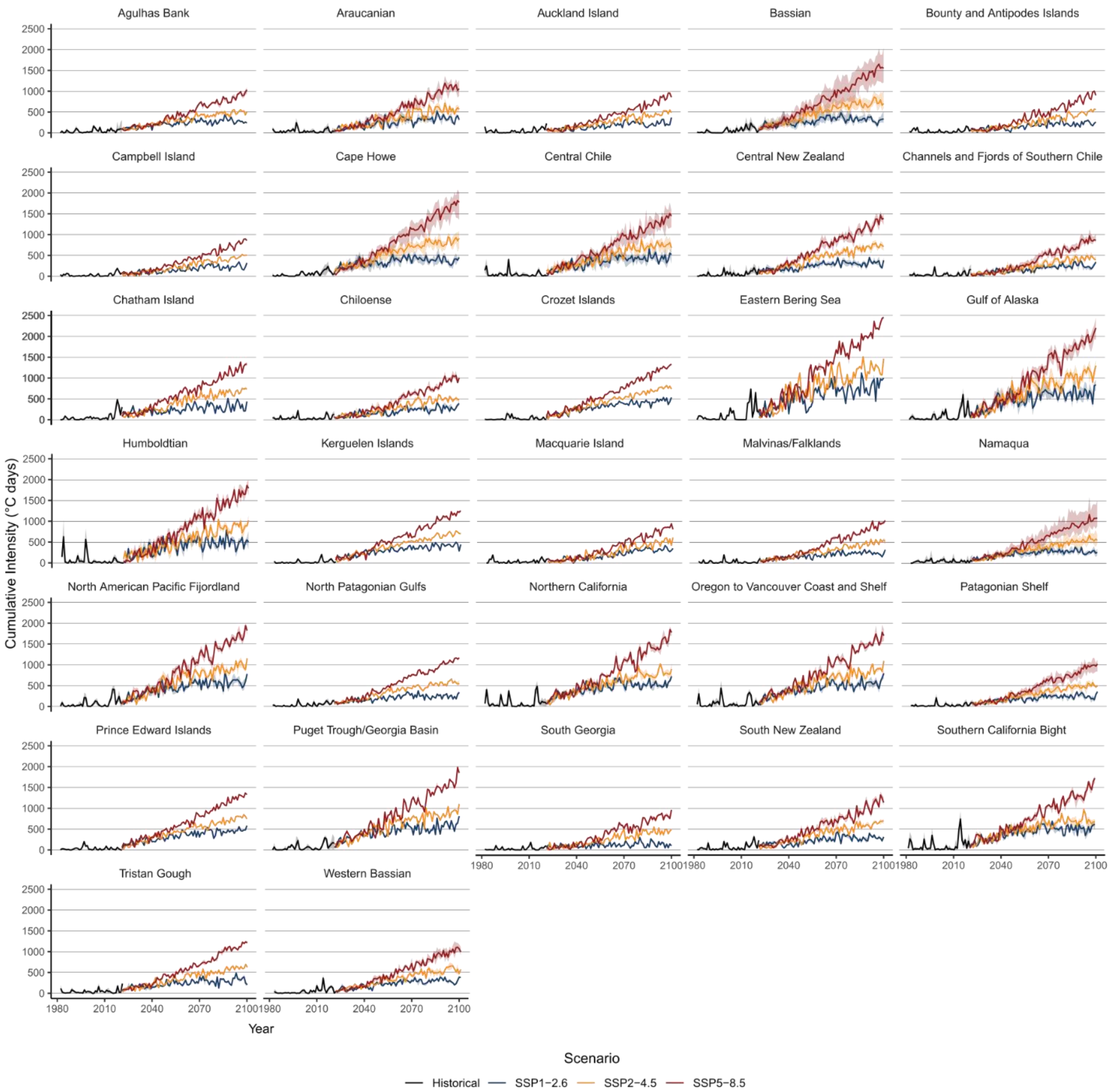

Supplementary Figure 3.- Ecoregional exposure of floating kelp forests to contemporary and future marine heatwaves. Ecoregion-specific exposure of kelp forest to historical (1982-2020) and future cumulative annual MHW intensities (2021-2100) across three climate scenarios (SSP-1.26, SSP-2.45, SSP-5.85). The solid line shows the mean across ensemble medians for all pixels, and the shaded area represents the 5th and 95th percentiles.

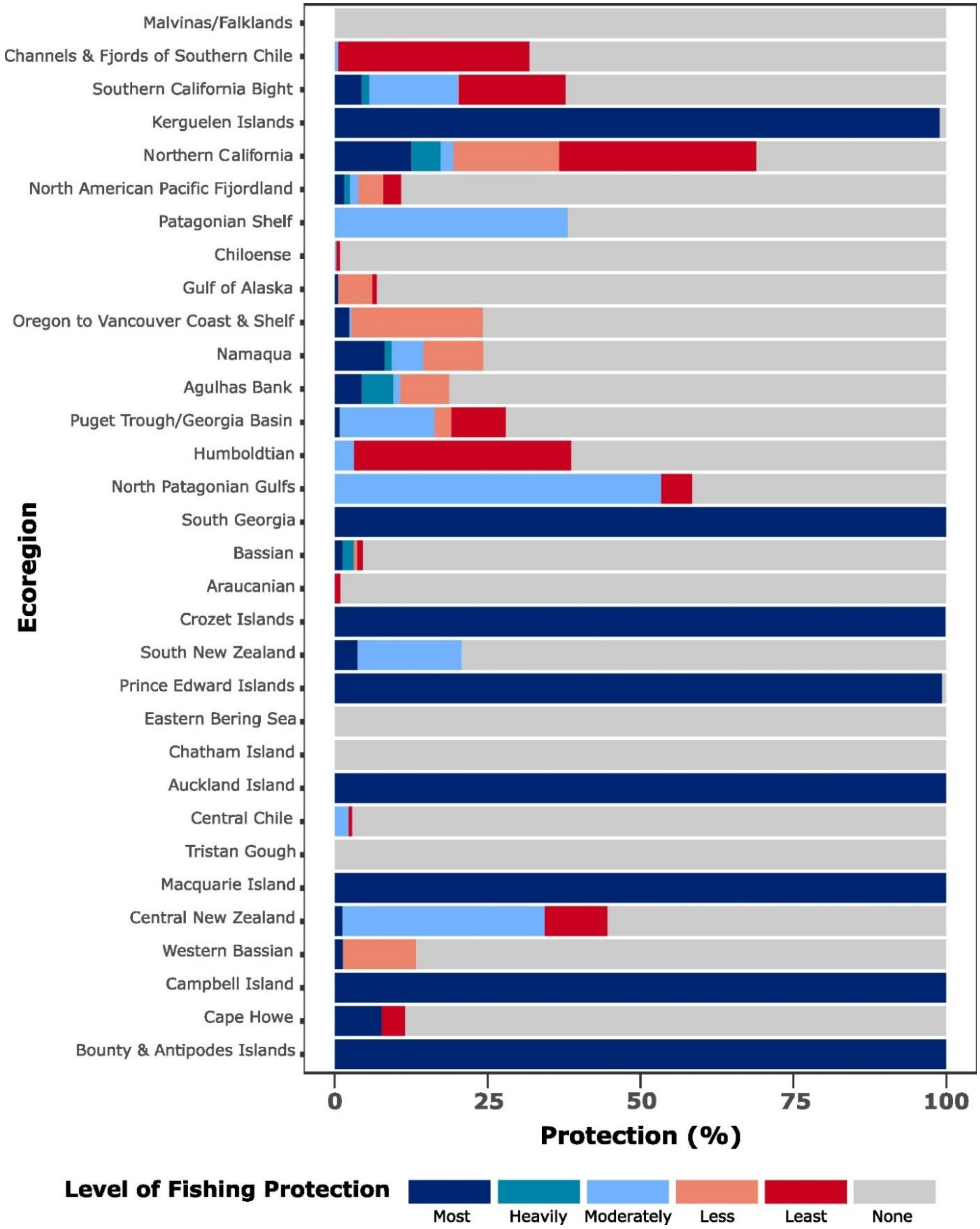

Supplementary Figure 4.- Global protection of floating kelp forests for each ecoregion. Percentage of kelp area protected in each ecoregion based on different levels of fishing restriction assigned to each marine protected area based on Protected Seas[1].

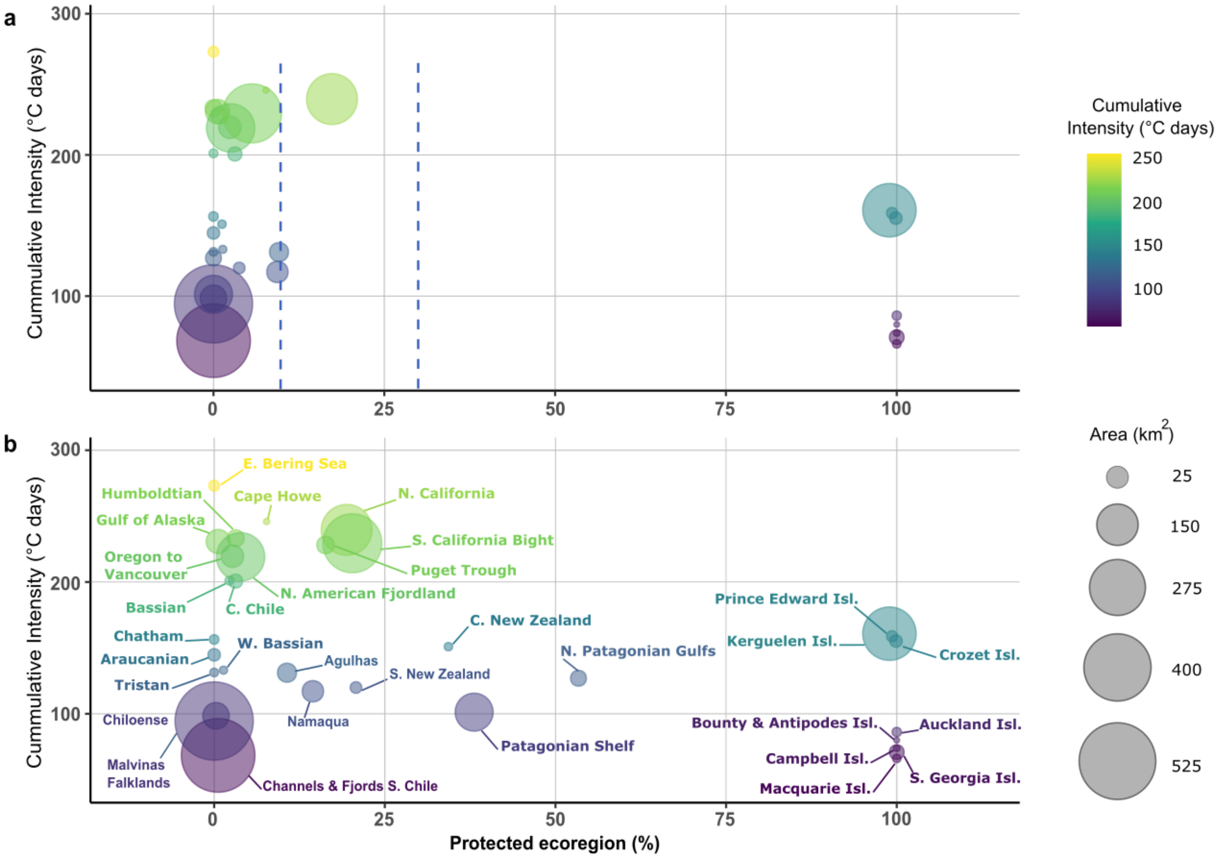

Supplementary Figure 5.- Relationship between threat posed by future marine heatwaves and level of protection. Scatterplots of mean future cumulative annual marine heatwave intensities for the near term (2021-2040) under SSP2-4.5 and the amount of kelp forest **a** highly protected, **b** highly and moderately protected combined. The size of the bubble indicates the amount of kelp in each ecoregion. The dashed blue vertical lines represent the old 10%[2] and the current 30%[3] targets for protection.

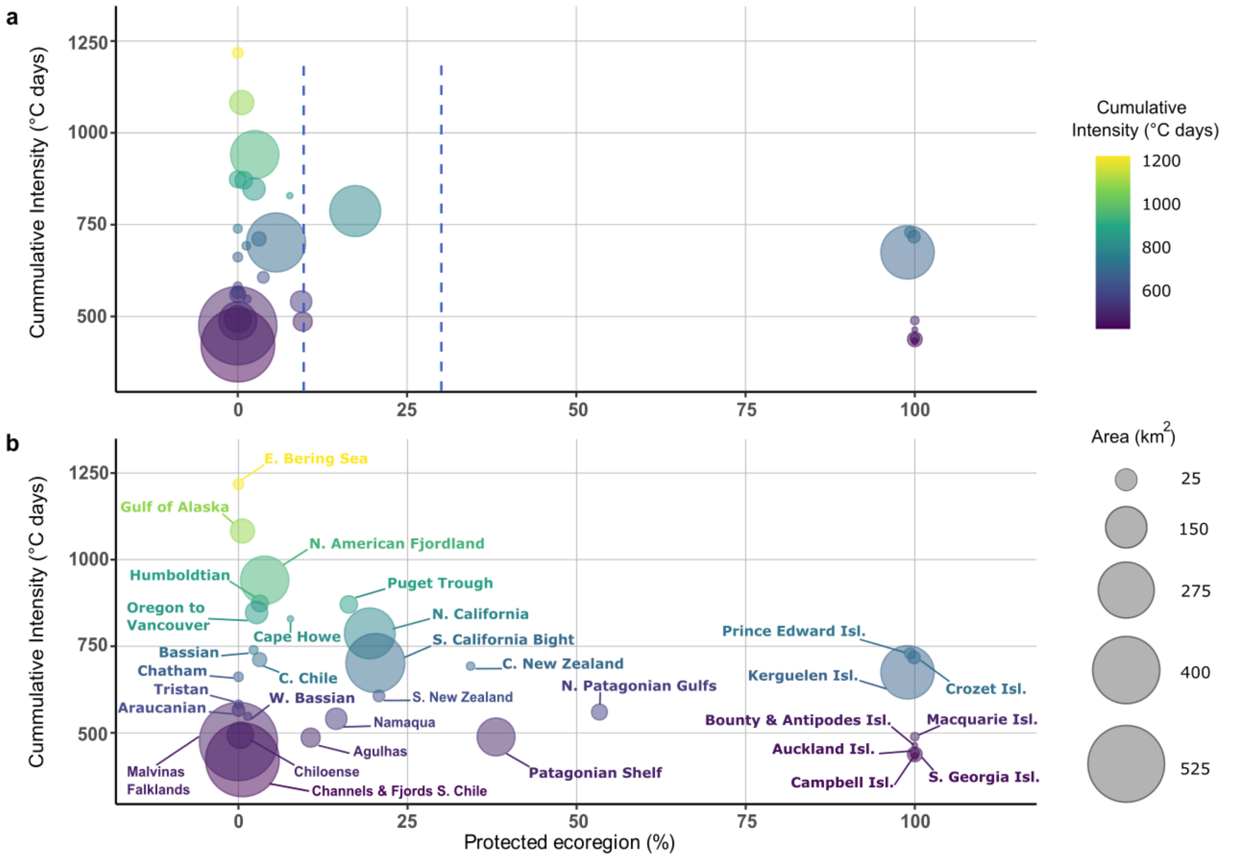

Supplementary Figure 6.- Relationship between threat posed by future marine heatwaves and level of protection. Scatterplots of mean future cumulative annual marine heatwave intensities for the long term (2081-2100) under SSP2-4.5 and the amount of kelp forest **a** highly protected, **b** highly and moderately protected combined. The size of the bubble indicates the amount of kelp in each ecoregion The dashed blue vertical lines represent the old 10%[2] and the current 30%[3] targets for protection.

Supplementary Table 1.- Data sources compiled to map the global distribution of floating kelp and marine protected areas (MPAs).

County	Landsat images	Sentinel-2 images	Coastline	MPAs
USA	California, Oregon, western Washington (including the outer coast and most of the Strait of Juan de Fuca), and southern Alaska. Landsat images from 1984-2022[4,5]	For eastern and northern Washington, from Puget Sound, San Juan Islands, and the Strait of Georgia, and northern Alaska. Sentinel-2 images from 2015-2019[6]	Alaska<br>https://catalog.northslopescience.org/dataset/851/resource/5986cd56-eb50-44e0-915c-ec9ad71b0d0b<br><br>Washington:<br>https://shoreline.noaa.gov/data/datasheets/medres.html	Government (NOAA)<br>https://marineprotectedareas.noaa.gov/dataanalysis/mpainventory/
Canada		For British Columbia. Sentinel-2 images from 2015-2019. Includes all British Columbia[6].	https://open.canada.ca/data/en/dataset/6c78fb2f-d23b-45b4-b3af-cc6f6cc4fff8	Government (CPCAD)<br>https://www.canada.ca/en/environment-climate-change/services/national-wildlife-areas/protected-conserved-areas-database.html
Mexico	All kelp maps for the country are from Landsat images for 1984-2022[4]		None	Government (CONANP)<br>http://sig.conanp.gob.mx/website/pagsig/info_shape.htm<br><br>Community-based MPAs and fishing refugees from COBI<br>https://cobi.org.mx/
Peru	All kelp maps for the country are from Landsat images for 1984-present[4]		None	Government (SERNANP)<br>https://geo.sernanp.gob.pe/visorsernanp/
Chile	East Chilean Antarctic Province from Isla Nueva (Long ~ -66.4) to the Beagle Channel in the west (Long ~-69) from latitude -55.5 to the Chile-Argentina international border. Landsat images for 1984-2022[4]	Most kelp maps for the country are based on Sentinel-2 images from 2015-2019[6].	https://www.marineregions.org/downloads.php	Government (SUBPESCA)<br>https://geoportal.subpesca.cl/portal/home/item.html?id=f1ece07b241740138fd07cb8404e328c
Argentina	All kelp maps for the country are from Landsat images for 1984-2022[4]		None	Government (IGN)<br>https://www.ign.gob.ar/NuestrasActividades/InformacionGeoespacial/CapasSIG

				WCS Argentina https://ampargentina.org/
Great Brittain	Falklands/Malvinas. Landsat images for 1984-2022[7]	For South Georgia and the South Sandwich Islands and Tristan da Cunha. Sentinel-2 images from 2015-2019[6]	Tristan da Cunha<br><br>https://www.marineregions.org/gazetteer.php?p=details&id=25511<br><br>South Georgia and the South Sandwich Islands<br><br>https://ramadda.data.bas.ac.uk/repository/entry/show?entryid=c1d83502-8799-4e3c-bdca-21db6a4405d4	MPA Atlas https://mpatlas.org/mpaguide/
Namibia		All kelp maps for the country are from Sentinel-2 images (2015-2019)[6]		MPA Atlas https://mpatlas.org/mpaguide/
South Africa		All kelp maps for continental South Africa are based on Landsat and Sentinel-2 images[8].<br><br>Prince Edwards Islands. Sentinel-2 images from 2015-2019[6]	Prince Edwards<br><br>https://marineregions.org/gazetteer.php?p=details&id=8384	Government (SAPAD)<br><br>https://egis.environment.gov.za/data_egis/data_download/current
France		All kelp maps for the country are from Sentinel-2 images (2015-2019)[6]	Crozet:<br>https://www.marineregions.org/gazetteer.php?p=details&id=25547<br>Kerguelen:<br>https://is.muni.cz/www/jaosobne/KergE.html	MPA Atlas https://mpatlas.org/mpaguide/
Australia	Tasmania coastline. Landsat images from 1986-2015[9]	South Australia, Victoria, and Macquarie Island.<br>Sentinel-2 images from 2015-2019[6]	South Australia and Victoria<br><br>https://ecat.ga.gov.au/geonetwork/srv/eng/catalog.search#/metadata/61395<br><br>Macquarie Island<br>https://data.gov.au/dataset/ds-dga-e4c01f74-de78-4233-80f4-444dfdc7380b/details	Government (CAPAD)<br>https://www.environment.gov.au/fed/catalog/search/resource/details.page?uuid=%7BAF4EE98E-7F09-4172-B95E-067AB8FA10FC%7D<br><br>Government Tasmania<br>https://listdata.thelist.tas.gov.au/opendata/index.html#LIST_Marine_Nature_Reserves
New Zealand		All kelp maps for the country are from Sentinel-2 images (2015-2019)[6]	https://data.linz.govt.nz/data/?q=coastline	https://data.linz.govt.nz/layer/53564-protected-areas/

Supplementary Table 2.- Mean historical cumulative annual marine heatwave intensities (C° days) (± 95% confidence intervals) for the contemporary term (2021-2040), mid (2041-2060), and long term (2081-2100) based on OISST historical data.

Realm	Ecoregion	Contemporary
Arctic	Eastern Bering Sea	177.4 ± 15.1
Temperate North Pacific	Gulf of Alaska	136.7 ± 1.8
	North American Pacific Fjordland	106.8 ± 1.1
	Northern California	81.1 ± 2.2
	Oregon to Vancouver Coast & Shelf	78.3 ± 3.3
	Puget Trough/Georgia Basin	80.5 ± 6.2
	Southern California Bight	118.8 ± 3.5
Temperate South America	Araucanian	36.9 ± 1.9
	Central Chile	35.8 ± 3.76
	Channels & Fjords of Southern Chile	41.1 ± 1.2
	Chiloense	40.0 ± 1.3
	Humboldtian	30.1 ± 1.5
	Falkland Islands (Malvinas)	39.5 ± 0.9
	North Patagonian Gulfs	20.5 ± 0.7
	Patagonian Shelf	31.3 ± 2.7
	Tristan Gough	43.4 ± 2.2
Southern Ocean	Auckland Island	55.2 ± 3.7
	Bounty & Antipodes Islands	43.6 ± 4.3
	Campbell Island	44.3 ± 1.1
	Crozet Islands	25.28 ± 0.5
	Kerguelen Islands	39.5 ± 0.9
	Macquarie Island	43.5 ± 6.3
	Prince Edward Islands	23.7 ± 4.3
	South Georgia	35.7 ± 0.8
Temperate Southern Africa	Agulhas Bank	60.9 ± 5.9
	Namaqua	44.8 ± 3.8
Temperate Australasia	Bassian	90.5 ± 5.6
	Cape Howe	89.9 ± 18.6
	Central New Zealand	68.7 ± 4.3
	Chatham Island	93.9 ± 5.4
	South New Zealand	78.7 ± 1.2
	Western Bassian	85.1 ± 2.8

Supplementary Table 3.- Mean future cumulative annual marine heatwave intensities (C° days) (± 95% confidence intervals) for the near (2021-2040), mid (2041-2060), and long term (2081-2100) under SSP1-2.6.

Realm	Ecoregion	Near-term SSP1.2-6	Mid-term SSP1.2-6	Long-term SSP1.2-6
Arctic	Eastern Bering Sea	299.6 ± 5.4	533.6 ± 6.9	786.7 ± 16.5
Temperate North Pacific	Gulf of Alaska	265.7 ± 1.5	476.0 ± 2.4	628.6 ± 4.7
	North American Pacific Fjordland	247.7 ± 1.0	440.3 ± 1.6	578.0 ± 1.5
	Northern California	253.4 ± 4.6	442.3 ± 3.8	537.5 ± 1.0
	Oregon to Vancouver Coast & Shelf	250.8 ± 5.1	418.2 ± 2.5	554.6 ± 4.0
	Puget Trough/Georgia Basin	258.8 ± 0.9	427.3 ± 0.6	569.6 ± 1.1
	Southern California Bight	224.9 ± 1.2	430.9 ± 3.7	507.4 ± 1.0
Temperate South America	Araucanian	120.9 ± 4.3	225.6 ± 8.2	346.4 ± 5.5
	Central Chile	178.4 ± 7.1	329.1 ± 17.3	447.5 ± 13.4
	Channels & Fjords of Southern Chile	75.3 ± 0.9	132.3 ± 0.9	258.2 ± 0.8
	Chiloense	98.6 ± 1.4	145.9 ± 2.6	273.9 ± 3.3
	Humboldtian	204.8 ± 6.5	425.1 ± 6.7	505.0 ± 8.6
	Falkland Islands (Malvinas)	97.7 ± 0.9	155.4 ± 0.8	245.3 ± 1.0
	North Patagonian Gulfs	117.3 ± 0.6	185.0 ± 1.1	243.2 ± 1.9
	Patagonian Shelf	107.9 ± 3.9	171.1 ± 2.6	250.5 ± 4.1
	Tristan Gough	142.6 ± 18.3	221.4 ± 14.7	310.6 ± 14.2
Southern Ocean	Auckland Island	88.4 ± 0.6	132.1 ± 3.0	219.3 ± 5.0
	Bounty & Antipodes Islands	93.5 ± 4.6	127.2 ± 5.8	220.0 ± 9.1
	Campbell Island	84.4 ± 1.1	125.5 ± 0.9	226.0 ± 2.1
	Crozet Islands	155.3 ± 1.9	284.5 ± 0.9	440.8 ± 1.5
	Kerguelen Islands	167.9 ± 1.4	288.3 ± 2.0	420.8 ± 2.7
	Macquarie Island	77.63 ± 3.4	141.9 ± 4.4	328.0 ± 8.9
	Prince Edward Islands	180.03 ± 1.1	305.1 ± 1.7	463.4 ± 2.8
	South Georgia	88.53 ± 0.9	97.3 ± 2.0	131.1 ± 2.3
Temperate Southern Africa	Agulhas Bank	131.3 ± 4.9	224.2 ± 3.4	287.0 ±1.4
	Namaqua	136.5 ± 5.3	230.1 ± 4.5	277.3 ± 3.5
Temperate Australasia	Bassian	211.9 ± 18.4	250.4 ± 16.5	312.7 ± 10.0
	Cape Howe	261.0 ± 46.7	327.4 ± 49.0	383.7 ± 41.5
	Central New Zealand	160.5 ± 5.0	222.4 ± 4.3	310.1 ± 3.6
	Chatham Island	177.9 ± 4.3	204.3 ± 2.0	311.6 ± 1.7
	South New Zealand	137.6 ± 2.7	188.1 ± 3.9	285.4 ± 1.5
	Western Bassian	124.2 ± 5	204.1 ± 3.7	310.3 ± 1.8

Supplementary Table 4.- Mean future cumulative annual marine heatwave intensities (C° days) (± 95% confidence intervals) for the near (2021-2040), mid (2041-2060), and long term (2081-2100) under SSP2-4.5.

**Realm**	**Ecoregion**	**Near-term SSP2.4-5**	**Mid-term SSP2.4-5**	**Long-term SSP2.4-5**
Arctic	Eastern Bering Sea	273.1 ± 7.0	752.4 ± 20.5	1218.2 ± 17.1
Temperate North Pacific	Gulf of Alaska	230.8 ± 1.5	632.7 ± 4.1	1082.6 ± 6.8
	North American Pacific Fjordland	219.1 ± 1.0	576.8 ± 2.3	940.5 ± 3.6
	Northern California	239.6 ± 1.6	499.7 ± 2.3	787.0 ± 7.5
	Oregon to Vancouver Coast & Shelf	219.5 ± 2.6	513.7 ± 8.4	847.4 ± 6.2
	Puget Trough/Georgia Basin	228.1 ± 0.5	540.6 ± 0.5	870.8 ± 0.3
	Southern California Bight	229.4 ± 2.3	479.6 ± 6.0	701.5 ± 13.0
Temperate South America	Araucanian	144.7 ± 4.7	279.4 ± 7.7	565.8 ± 12.6
	Central Chile	201.1 ± 7.5	392.7 ± 19.0	739.0 ± 23.5
	Channels & Fjords of Southern Chile	68.5 ± 1.2	173.9 ± 2.0	422.0 ± 2.6
	Chiloense	98.5 ± 1.6	219.4 ± 3.0	493.2 ± 2.2
	Humboldtian	233.2 ± 7.1	436.0 ± 8.5	873.5 ± 3.3
	Falkland Islands (Malvinas)	94.5 ± 0.8	241.2 ± 1.0	475.0 ± 1.3
	North Patagonian Gulfs	127.0 ± 1.4	304.3 ± 2.9	559.9 ± 0.9
	Patagonian Shelf	101.3 ± 5.2	258.6 ± 8.5	488.1 ± 10.8
	Tristan Gough	131.4 ± 4.1	288.8 ± 12.0	582.3 ± 14.6
Southern Ocean	Auckland Island	86.2 ± 1.9	208.1 ± 2.8	441.2 ± 6.4
	Bounty & Antipodes Islands	80.0 ± 2.1	195.8 ± 8.8	464.0 ± 13.9
	Campbell Island	73.9 ± 0.1	182.6 ± 2.2	432.4 ± 2.9
	Crozet Islands	155.2 ± 2.3	361.3 ± 1.9	717.4 ± 2.2
	Kerguelen Islands	160.8 ± 1.2	369.4 ± 2.2	674.7 ± 2.7
	Macquarie Island	66.2 ± 6.2	175.5 ± 5.8	488.9 ± 11.2
	Prince Edward Islands	158.9 ± 0.4	383.9 ± 2.1	729.2 ± 3.9
	South Georgia	70.9 ± 0.9	192.4 ± 3.7	437.8 ± 4.2
Temperate Southern Africa	Agulhas Bank	131.2 ± 2.5	276.0 ± 6.5	486.0 ± 9.3
	Namaqua	117.1 ± 3.1	294.5 ± 9.9	540.6 ± 23.4
Temperate Australasia	Bassian	200.7 ± 16.2	399.2 ± 21.9	711.3 ± 25.9
	Cape Howe	245.9 ± 37.3	487.5 ± 64.9	828.9 ± 68.6
	Central New Zealand	151.0 ± 2.6	356.7 ± 5.7	692.6 ± 9.1
	Chatham Island	156.4 ± 3.0	329.9 ± 1.0	661.7 ± 5.3
	South New Zealand	119.9 ± 2.1	295.9 ± 4.2	606.5 ± 5.3
	Western Bassian	133.1 ± 4.3	284.9 ± 10.9	547.4 ± 20.6

Supplementary Table 5.- Mean future cumulative annual marine heatwave intensities (C° days) (± 95% confidence intervals) for the near (2021-2040), mid (2041-2060), and long term (2081-2100) under SSP5-8.5.

Realm	Ecoregion	Near-term SSP5.8-5	Mid-term SSP5.8-5	Long-term SSP5.8-5
Arctic	Eastern Bering Sea	358.9 ± 26.5	870.4 ± 21.4	2042.8 ± 17.8
Temperate North Pacific	Gulf of Alaska	283.7 ± 2.5	718.8 ± 6.7	1830.9 ± 9.1
	North American Pacific Fjordland	280.8 ± 0.8	660.1 ± 2.1	1588.3 ± 3.9
	Northern California	276.9 ± 2.5	582.0 ± 1.6	1469.2 ± 11.4
	Oregon to Vancouver Coast & Shelf	278.6 ± 4.6	612.4 ± 9.6	1504.1 ± 10.5
	Puget Trough/Georgia Basin	291.0 ± 0.9	640.1 ± 1.3	1535.8 ± 1.3
	Southern California Bight	259.6 ± 2.8	574.7 ± 4.1	1352.8 ± 13.4
Temperate South America	Araucanian	122.4 ± 5.6	361.8 ± 13.7	1019.8 ± 14.7
	Central Chile	207.1 ± 11.9	538.5 ± 18.2	1269.4 ± 38.8
	Channels & Fjords of Southern Chile	71.8 ± 1.1	225.1 ± 2.4	764.9 ± 5.1
	Chiloense	94.6 ± 0.8	292.1 ± 3.1	886.0 ± 3.8
	Humboldtian	186.9 ± 8.6	624.5 ± 6.3	1551.1 ± 11.6
	Falkland Islands (Malvinas)	98.3 ± 1.3	276.1 ± 1.6	835.5 ± 4.1
	North Patagonian Gulfs	139.7 ± 0.6	404.1 ± 2.1	1014.8 ± 2.6
	Patagonian Shelf	105.8 ± 4.2	316.3 ± 11.3	874.0 ± 22.7
	Tristan Gough	149.7 ± 6.3	423.0 ± 15.6	1081.6 ± 6.0
Southern Ocean	Auckland Island	92.9 ± 1.8	284.9 ± 3.2	753.4 ± 5.7
	Bounty & Antipodes Islands	88.9 ± 3.9	279.3 ± 4.2	791.2 ± 15.8
	Campbell Island	89.7 ± 1.1	256.9 ± 4.4	722.4 ± 7.5
	Crozet Islands	176.1 ± 2.9	472.0 ± 1.0	1155.7 ± 2.4
	Kerguelen Islands	160.9 ± 1.6	468.1 ± 1.5	1089.4 ± 4.7
	Macquarie Island	77.1 ± 3.8	217.1 ± 11.3	742.7 ± 15.8
	Prince Edward Islands	203.7 ± 4.6	486.2 ± 1.7	1194.7 ± 7.8
	South Georgia	68.1 ± 1.9	253.1 ± 3.2	770.3 ± 6.4
Temperate Southern Africa	Agulhas Bank	140.3 ± 4.2	337.7 ± 11.3	838.2 ± 11.3
	Namaqua	152.9 ± 5.8	379.5 ± 18.3	959.6 ± 49.9
Temperate Australasia	Bassian	193.9 ± 16.2	551.1 ± 30.5	1405.4 ± 62.8
	Cape Howe	223.3 ± 37.0	623.2 ± 60.3	1583.4 ± 137.5
	Central New Zealand	174.2 ± 3.2	496.1 ± 7.7	1194.4 ± 15.2
	Chatham Island	165.0 ± 2.8	508.3 ± 3.1	1147.5 ± 6.8
	South New Zealand	144.6 ± 3.3	416.2 ± 6.5	1013.5 ± 12.1
	Western Bassian	109.9 ± 7.5	368.0 ± 15.6	956.9 ± 35.3

2.34558206 4.02161894 5.6273492 2.16590087 5.30689632 9.60452843 2.37822995 6.14745771 16.555539

Supplementary Table 6.- Climate models with daily sea surface temperature used in this study.

Modelling Center	Institute ID	Model Name
Commonwealth Scientific and Industrial Research Organisation, Australia	CSIRO	ACCESS-CM2<br>ACCESS-ESM1
Beijing Climate Center, China Meteorological Administration	BC	BCC-CSM2-MR
Canadian Centre for Climate Modelling and Analysis	CCCMA	CanESM5
Centre National de Recherches Meteorologiques/ Centre Europeen de Recherche et Formation Avancees en Calcul Scientifique	CNRM-CERFACS	CNRM-CM6-1<br>CNRM-ESM2-1
EC-Earth consortium	EC-Earth	EC-Earth3
Atmosphere and Ocean Research Institute (The University of Tokyo), National Institute for Environmental Studies, and Japan Agency for Marine -Earth Science and Technology	MIROC	MIROC6
Meteorological Research Institute	MRI	MRI-ESM2-0
Norwegian Climate Center	NCC	NorESM2-LM<br>NorESM2-MM

References

1 Driedger, A. *et al.* Guidance on marine protected area protection level assignments when faced with unknown regulatory information. *Marine Policy* **148**, 105441 (2023).

2 CBD. Strategic Plan for Biodiversity 2011–2020, Convention on Biological Diversity. Montreal., (2010).

3 Diversity, C. o. B. (Convention on Biological Diversity Montreal).

4 Bell, T. W. *et al.* Kelpwatch: A new visualization and analysis tool to explore kelp canopy dynamics reveals variable response to and recovery from marine heatwaves. *PLoS One* **18**, e0271477 (2023).

5 McPherson, M. L. *et al.* Large-scale shift in the structure of a kelp forest ecosystem co-occurs with an epizootic and marine heatwave. *Communications biology* **4**, 1-9 (2021).

6 Mora-Soto, A. *et al.* A high-resolution global map of Giant kelp (Macrocystis pyrifera) forests and intertidal green algae (Ulvophyceae) with Sentinel-2 imagery. *Remote Sensing* **12**, 694 (2020).

7 Houskeeper, H. F. *et al.* Automated satellite remote sensing of giant kelp at the Falkland Islands (Islas Malvinas). *PLoS One* **17**, e0257933 (2022).

8 Dunga, V. L. Mapping and assessing ecosystem threat status of South African kelp forests. (2020).

9 Butler, C. L., Lucieer, V. L., Wotherspoon, S. J. & Johnson, C. R. Multi-decadal decline in cover of giant kelp Macrocystis pyrifera at the southern limit of its Australian range. *Marine Ecology Progress Series* **653**, 1-18 (2020).
